# Isolation and Phylogenetic Grouping of Equine Encephalosis Virus in Israel

**DOI:** 10.3201/eid1710.110350

**Published:** 2011-10

**Authors:** Karin Aharonson-Raz, Amir Steinman, Velizar Bumbarov, Sushila Maan, Narender Singh Maan, Kyriaki Nomikou, Carrie Batten, Christiaan Potgieter, Yuval Gottlieb, Peter Mertens, Eyal Klement

**Affiliations:** The Hebrew University, Rehovot, Israel (K. Aharonson-Raz, A. Steinman, Y. Gottlieb, E. Klement);; Kimron Veterinary Institute, Bet Dagan, Israel (V. Bumbarov);; Institute for Animal Health, Pirbright, UK (S. Maan, N.S. Maan, K. Nomikou, C. Batten, P. Mertens);; Deltamune (Pty) Ltd, Lyttelton, South Africa (C. Potgieter)

**Keywords:** equine encephalosis virus, EEV, arboviruses, orbivirus, horse, African horse sickness virus, African horse sickness, Culicoides, virus, dispatch

## Abstract

During 2008–2009 in Israel, equine encephalosis virus (EEV) caused febrile outbreaks in horses. Phylogenetic analysis of segment 10 of the virus strains showed that they form a new cluster; analysis of segment 2 showed ≈92% sequence identity to EEV-3, the reference isolate. Thus, the source of this emerging EEV remains uncertain.

Equine encephalosis is an arthropod-borne, noncontagious, febrile disease of horses. It was first described >100 years ago by A. Theiler (*1*) under the name equine ephemeral fever. The disease is caused by *Equine encephalosis virus* (EEV; genus *Orbivirus*: subfamily *Sedoreovirinae*: family *Reoviridae)* (*2**,**3*), which is transmitted by *Culicoides* spp. biting midges (*4*). Before 2008, EEV had been isolated only in South Africa, where 7 antigenically distinct serotypes, EEV-1–7, have been identified and characterized (*3*).

Orbiviruses encode at least 7 structural and 4 nonstructural (NS) proteins from 10 linear dsRNA genome segments (*5*). The smallest genome segment, segment 10 (Seg-10), encodes NS3, which mediates the release of virus particles from infected cells, and NS3A. The second largest of the EEV genome segments, Seg-2, encodes virus protein (VP) 2, the larger of the 2 outer-capsid proteins. By analogy with bluetongue virus (BTV), the *Orbivirus* type species, the virus serotype is determined by the specificity of interactions between VP2 and neutralizing antibodies generated during infection of the mammalian host. Consequently, VP2 and Seg-2 show sequence variations that correlate with serotype and, thus, can be used to determine the virus serotype (*6*).

From October 2008 through January 2009, a febrile horse disease that was diagnosed as equine encephalosis was observed in dozens of stables across Israel (*7*). The recent emergence of novel orbivirus strains (including BTV and epizootic hemorrhagic disease virus) in Europe, North America, Asia, and Australia (*8*) is of major concern to the worldwide livestock industry. Furthermore, the similarity of EEV to African horse sickness virus, one of the most devastating pathogens of equids, warranted further investigation of the outbreaks and molecular characterization of the virus. The molecular and sequence analyses reported here confirm the existence of EEV in Israel and identify the virus and its serotype, as well as its phylogenetic roots.

## The Study

**Figure 1:**
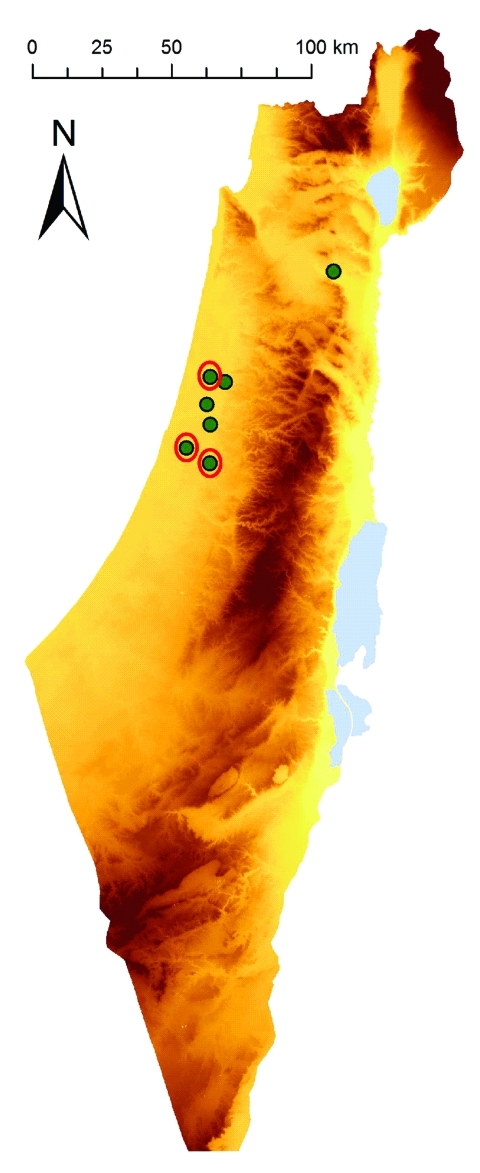
Geographic location of farms in Israel with horses showing signs of equine encephalosis virus (EEV) infection. Eight horses with suspected EEV infection lived on 7 farms. Red circles indicate farms with EEV-positive cases.

During October–November 2009, samples of whole blood from 8 febrile horses (H1–8; temperatures 39.5°C–42°C) in Israel were collected into EDTA tubes and analyzed at the Koret School of Veterinary Medicine (Hebrew University, Rehovot, Israel). Vero cell (American Type Culture Collection, Manassas, VA, USA) culture results of blood from H3, H5, and H8 were positive for EEV (Table 1; Figure 1).

**Table 1:** Clinical signs for horses whose blood was tested to determine the cause of a febrile disease, Israel, October–November 2009

Horse no.	Clinical signs	Date of first clinical sign	Duration of clinical signs, d	Date of blood collection	Virus isolated*
1	Temperature 39.5°C, lack of appetite	Oct 25	5	Nov 2	No
2	Temperature 39.5°C, lack of appetite	Oct 29	3	Nov 2	No
3	Temperature 40°C, lack of appetite	Oct 30	3	Nov 3	Yes
4	Temperature 39.5°C, lack of appetite	Nov 8	Unknown	Nov 8	No
5	Fever, colic, lethargy, congested mucous membranes, rapid pulse, lack of appetite	Nov 5	5	Nov 9	Yes
6	Temperature 39.5°C, lack of appetite	Nov 17	4	Nov 22	No
7	Temperature 42°C, apathy	Nov 25	2	Nov 26	No
8	Temperature 39.7°C, lack of appetite, colic	Nov 26	3	Nov 27	Yes

*Positive cases were confirmed by reverse transcription PCR of dsRNA genome segment 10.

Total RNA was extracted from the fifth and sixth passages of all 3 samples by using the QIAamp Viral RNA Mini Kit (QIAGEN, Valencia, CA, USA) to obtain sufficient viral load for the subsequent analyses and replications. RNA was reverse transcribed into cDNA by using the Verso cDNA Kit (Thermo Fisher Scientific, Epsom, UK). PCR amplification of the gene encoding NS3 (Seg-10) was performed on the 3 isolates by using GoTaq Green Master Mix (Promega, Madison, WI, USA) with the following primers: 5′-^1^GTT AAG TTT CTG CGC CAT GT^23^-3′, 5′-^741^GTA ACA CGT TTC CGC CAC G^760^-3′. Thermal cycling conditions for the PCR were as previously described (*9*); the primer annealing temperature was modified to 53.5°C. PCR products were purified by using a cDNA purification kit (ExoSAP-IT; USB, Cleveland, OH, USA), and sequencing was conducted by BigDye terminator cycle sequencing chemistry (Applied Biosystems, Foster City, CA, USA) in an ABI 3700 DNA Analyzer (Applied Biosystems) by using ABI data collection and sequence analysis software. Further analysis of the NS3 sequence was performed with Sequencer software, version 4.8 (Gene Codes Corp., Ann Arbor, MI, USA). Sequences were deposited in GenBank under accession nos. HQ441245 for H5, HQ441246 for H3, and HQ441247 for H8. The NS3 genes (Seg-10) were compared with those of different EEVs (*9*) and other related orbiviruses (Table 2). Phylogenic trees were generated by using the neighbor-joining and maximum likelihood methods (Phylip Inference Package version 3.68, Seqboot Program; J. Felsenstein, University of Washington, Seattle, WA, USA) to create 100 datasets (bootstrapping) and the DNA Maximum Likelihood Program version 3.5 (http://cmgm.stanford.edu/phylip/dnaml.html) to construct the trees. Finally, the Consense program version 3.5c (http://cmgm.stanford.edu/phylip/consense.html) was used to create a final consensus tree for our dataset. Broadhaven virus, a tick-borne orbivirus, was used as the outgroup in the phylogram for the gene encoding NS3.

**Table 2:** GenBank accession numbers for orbiviruses used for phylogenic analyses of strain isolated from horses in Israel, 2009*

Virus	Strain	Accession no.	
		Seg-2	Seg-10
EEV-1 (Cascara)	FLD1		AY115878
	FLD2		AY115876
	FLD3		AY115875
	FLD4		AY115877
	Ref		AY115865
	Ref†		AY115864
EEV-2 (Gamil)	Ref		AY115871
EEV-3 (Kaalplaas)	FLD1		AY115874
	Ref	HQ630933	AY115867
EEV-4 (Bryanston)	Ref		AY115868
EEV-5 (Kyalami)	Ref		AY115869
EEV-6 (Potchefstroom)	Ref		AY115866
	FLD1		AY115872
	FLD2		AY115873
EEV-7 (N Rand)	Ref		AY115870
AHSV-2			AF276700
AHSV-4			AJ007305
AHSV-7			AJ007306
BRDV			M83197
BTV- 2			AF135224
BTV-12			AF135227
PALV (Chuzan)			AB018091
EHDV-1			NC_013405.1
EHDV-2			AM745086.1
Israel EEV H5	Animal H5		HQ441245
Israel EEV H3	Animal H3 (ISR2009/20)‡	JF495411	HQ441246
Israel EEV H8	Animal H8 (ISR2009/21)‡	JF495412	HQ441247

*Seg, dsRNA genome segment; EEV, equine encephalosis virus; FLD, field strain; ref, reference strain; N Rand, North Rand; AHSV, African horse sickness virus; BRDV, Broadhaven virus; BTV, bluetongue virus; PALV, Palyam virus; EHDV, epizootic hemorrhagic disease virus; H5, H3, H8, horses 5, 3, 8. †Unknown origin. ‡Isolate numbers for the *Orbivirus* reference collection (www.reoviridae.org/dsRNA_virus_proteins/ReoID/EEV-isolates.htm).

**Figure 2:**
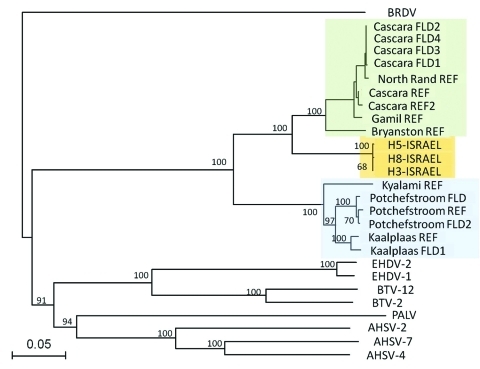
Phylogeny of equine encephalosis virus (EEV) segment 10 (nonstructural protein 3 gene) isolated from horses in Israel in 2009. The phylogenic tree was constructed by using the neighbor-joining method and bootstrapped with 100 replicates. Branch lengths are indicative of the genetic distances between sequences. Other orbiviruses were included for reference, with Broadhaven virus (BRDV) selected as the outgroup. The 3 suggested EEV clusters are marked green (cluster A), blue (cluster B), and orange (cluster C, representing the isolates from Israel). REF, reference; FLD, field strain; H3, H5, H8, horse 3, 5, and 8; EHDV, epizootic hemorrhagic disease virus; BTV, bluetongue virus; PALV, Palyam virus; AHSV, African horse sickness virus. Scale bar indicates nucleotide substitution per site.

The phylogenetic analyses of EEV Seg-10 grouped the Israeli isolates with other EEV isolates but as a distinct group with no close relation to African horse sickness virus, BTV, or epizootic hemorrhagic disease virus. Within the EEV group, 3 discrete clusters (A, B, C) were recognized; the Israeli isolates formed one of these clusters (C; Figure 2). The Israeli isolates have 85%–86% nt identity to cluster A and 75%–76% nt identity to cluster B.

In addition, full-length cDNA copies of individual EEV (from H3 and H8) genome segments were synthesized and amplified by reverse transcription PCR by using the anchor spacer–ligation method as described (*10**,**11*). Partial sequences (for the upstream 450 bp) of Seg-2 from the different Israeli isolates were identical, showing 92.3% nt and 95.7% aa sequence identity with Seg-2 and VP2 of the Kaalplaas isolate, the reference isolate of EEV-3 (GenBank accession numbers are listed in Table 2). Previous phylogenetic comparisons of Seg-2/VP2 from different BTV types showed a maximum of 71% nt and 78% aa acid identity between serotypes (*6*), indicating that the isolates from Israel also belong to EEV type 3.

## Conclusions

Equine encephalosis virus has long been enzootic to southern Africa, but it has not been isolated in other parts of the world. We report the characterization of an EEV strain isolated outside of Africa. Phylogenetic analysis of Seg-2 showed 92% sequence identity to EEV-3 (Kaalplaas).

Analysis of Seg-10 (the gene encoding NS3) of different orbiviruses showed 2 clusters of South African EEV strains (A and B), in agreement with previously published studies (*9*). These 2 clusters appear to correlate with the geographic origins of the viruses in South Africa, independent of their isolation date. It has been suggested that the 2 EEV Seg-10 clusters in South Africa are related to the distribution of their *Culicoides* spp. midge vectors, *C. imicola* (*senso stricto*) and *C. bolitinos*. The former is the most abundant *Culicoides* spp. midge in Israel (*12*). However, the EEV isolates from Israel group as a distinct cluster (C) with similar distances to the 2 South African clusters, raising questions concerning the geographic origin of this virus. A similar finding has been observed in African horse sickness virus Seg-10, which also forms into 3 distinct groups (*13*).

The question of how and when the virus was initially introduced to Israel remains unanswered. Because the clinical manifestations of equine encephalosis are usually mild, it is often overlooked and underdiagnosed. EEV could have been introduced to Israel before the virus was first isolated in 2009. Alternatively, the virus might have been introduced into neighboring countries and transmitted into Israel by infected vectors carried by winds, as described for other orbiviruses (*14**,**15*). The fact that the Israeli strain of EEV-3 grouped in a different cluster than the 2 South African strains, supports the idea that it has evolved in this region for a sufficient time to accumulate these changes and most likely was not recently introduced into Israel from South Africa.
